# Wheat leaf dark respiration acclimates more strongly at night than in the day when responding to nocturnal warming

**DOI:** 10.1093/jxb/erag106

**Published:** 2026-02-26

**Authors:** Pratima Rana Shahi, Andrew P Scafaro, Rebecca J Thistlethwaite, Owen K Atkin, Richard M Trethowan, Romina Rader, Adrienne Burns, Onoriode Coast

**Affiliations:** School of Environmental and Rural Sciences, Faculty of Science, Agriculture, Business, and Law, University of New England, Armidale, NSW 2351, Australia; Division of Plant Science, Research School of Biology, The Australian National University, Canberra, ACT 2601, Australia; School of Life and Environmental Sciences, Plant Breeding Institute, Sydney Institute of Agriculture, The University of Sydney, Narrabri, NSW 2390, Australia; Division of Plant Science, Research School of Biology, The Australian National University, Canberra, ACT 2601, Australia; School of Life and Environmental Sciences, Plant Breeding Institute, Sydney Institute of Agriculture, The University of Sydney, Narrabri, NSW 2390, Australia; School of Life and Environmental Sciences, Plant Breeding Institute, Sydney Institute of Agriculture, The University of Sydney, Cobbitty, NSW 2570, Australia; School of Environmental and Rural Sciences, Faculty of Science, Agriculture, Business, and Law, University of New England, Armidale, NSW 2351, Australia; School of Environmental and Rural Sciences, Faculty of Science, Agriculture, Business, and Law, University of New England, Armidale, NSW 2351, Australia; School of Environmental and Rural Sciences, Faculty of Science, Agriculture, Business, and Law, University of New England, Armidale, NSW 2351, Australia; University of Nottingham, UK

**Keywords:** Acclimation, climate change, dark respiration, high night temperature, night respiration, photosynthetic efficiency, *Triticum aestivum*, warm nights, wheat

## Abstract

Rising night temperatures pose a significant threat to wheat productivity, yet the physiological basis of wheat adaptation to nocturnal warming remains poorly understood. We evaluated leaf photosynthetic and respiratory traits in response to warm nights under temperature-controlled environments in 10 Australian wheat cultivars released between 1901 and 2012. When exposed to warmer nights, rates of leaf net CO_2_ assimilation measured at 25 °C (*A*_net_^25^) remained stable across cultivar release date despite declines in photosynthetic capacity (*V*_cmax_ and *J*_1500_) in newer cultivars. In most cultivars leaf respiratory CO_2_ release in the dark (*R*_dark_) exhibited divergent thermal responses: warm nights suppressed temperature-normalised night *R*_dark_ (*R*_dark_night_) but stimulated or maintained *R*_dark_ in the daytime (*R*_dark_day_). The results suggest that a century of yield-focused breeding might have inadvertently maintained *A*_net_^25^ under warmer nights in modern cultivars. This most likely reflects the selection of genotypes with more efficient photosynthetic capacity (i.e. greater return per protein investment) under warm nights. It is also likely that modern cultivars exhibit reduced respiratory demand for maintenance of processes such as Rubisco protein turnover and synthesis. Our findings highlight trait-based targets for enhancing energy efficiency and climate resilience in wheat and opportunities to improve the parameterisation of *R*_dark_ in Earth system models.

## Teaser

Warm nights suppress night-time respiration in wheat without reducing photosynthetic capacity, contradicting the common assumption that warmer nights uniformly increase respiratory carbon loss.

## Introduction

Wheat is a staple food crop for about 3 billion people and provides 20% of human calorie and protein intake (Shewry and Hey, 2015). It is cultivated on more than 220 million ha worldwide (Ramirez-Villegas *et al*., 2020; Hay *et al*., 2022) and demand for wheat is expected to rise as the global population is predicted to increase to 9 billion by 2050 (Alexandratos and Bruinsma, 2012; Kinnunen *et al*., 2020). Meeting growing demands for wheat is challenging, and is compounded by human-induced climate change (Mondal, 2010; Poveda *et al*., 2018). Climate change has been marked by increased land-surface temperatures at global, regional, and national scales compared with pre-industrial levels. The increases in global day and night temperatures have been asymmetric, with night-time minimum temperature increasing up to 1.4-fold faster than day-time maximum temperatures at the global scale (Jones *et al*., 1999; Vose *et al*., 2005; Sillmann *et al*., 2013; Davy *et al*., 2017; Zhuang and Zhang, 2020). The rise in night temperatures has been attributed to cloud cover, radiative forcing induced by anthropogenic greenhouse gases, and vegetation feedbacks (or greening) that reduce radiative heat loss at night (Kaiser, 1998; Shi *et al*., 2021). Whilst trapping heat leaving the Earth’s surface at night, cloud cover also reduces the amount of incoming solar shortwave radiation during the day, which lessens daily maximum warming (Karl *et al*., 1984; Kukla and Karl, 1993). Other minor contributors are changes in land use, urban warming effects, and changes in soil moisture (Lough, 1995; Shi *et al*., 2021).

It is predicted that nocturnal warming will continue (Cox *et al*., 2020; J. Wang *et al*., 2020; Zhuang and Zhang, 2020; Doan *et al*., 2022) if anthropogenic greenhouse gas emissions are not mitigated. Despite night temperatures rising more rapidly than day temperatures, and the well-documented negative effects of warm nights on crop yield (as described below), research in crop thermal physiology has become increasingly asymmetrical with more studies focusing on responses to high day-time temperatures than to high night-time temperatures (Reynolds *et al*., 2021). Hot nights pose serious risks to ecosystem functions and services, and crop yields (Welch *et al*., 2010; Sadok and Jagadish, 2020). Night warming has been strongly linked with declines in wheat yields , more so than rising day temperatures (García *et al*., 2015; Fischer *et al*., 2022). A loss in grain yield of 2–9% °C^−1^ in wheat has been reported from field and modelling studies of daily minimum temperature in the USA and Mexico (Russell and Van Sanford, 2020; Fischer *et al*., 2022; McAusland *et al*., 2023). A similar response has been reported for rice, with grain yield declining by 10% for every 1 °C increase in growing-season minimum temperature (Peng *et al*., 2004). Cereal yield losses due to warm nights are probably due to disrupted reproductive processes (Coast *et al*., 2015; Narayanan *et al*., 2018), shortening of the critical development period for determination of grain yield (Fischer *et al*., 2024), increased respiratory CO_2_ losses during the night (García *et al*., 2016; Impa *et al*., 2019), and reduced capacity for carbon fixation through photosynthesis (Posch *et al*., 2019; Coast *et al*., 2024). Wheat leaf net CO_2_ assimilation (*A*_net_) and respiratory CO_2_ release (*R*_dark_), which contribute strongly to the total carbon budget and ultimately yield, are sensitive to temperature. In the short term, *A*_net_ increases with instantaneous temperature rises from sub-optimal levels, reaches a peak at an optimum temperature, and then declines with further warming (Posch *et al*., 2019). This curvilinear response reflects the impacts on photosynthetic CO_2_ fixation, including by reduced Rubisco fixation and electron transport capacity with rising temperature and CO_2_ release by photorespiration and *R*_dark_ (Yamori *et al*., 2014; Scafaro *et al*., 2023). Over long time-frames, warmer growth conditions can cause increases in the thermal optimum of *A*_net_ (Berry and Bjorkman, 1980; Way and Yamori, 2014). However, heat stress including warm nights can cause down-regulation of both *A*_net_ and photosynthetic capacity (i.e. the maximum carboxylation rate *V*_cmax_, and the maximum electron transport rate *J*_max_). For example, inhibition of photosynthesis under warm nights occurs in some wheat cultivars (Posch *et al*., 2019; Coast *et al*., 2024), and other crops such as maize (Sinsawat *et al*., 2004) cotton (Reddy *et al*., 1997), and soybean (Sankarapillai *et al*., 2025).

Like photosynthesis, *R*_dark_ responds to instantaneous and long-term temperature changes, but the response patterns vary depending on the time-frame being considered. In the short term, a rise in temperature induces a near-exponential increase in *R*_dark_ (Atkin and Tjoelker, 2003) up to a maximum at ∼50–60 °C (*T*_max_) beyond which the respiration rate abruptly declines (O’Sullivan *et al*., 2017). The quick rise in *R*_dark_ is linked to increased energy demand for upkeeping functions, and diversion of assimilated carbon away from growth (Scafaro *et al*., 2021). Beyond *T*_max_, the rapid decrease in *R*_dark_ probably reflects irreversible injury to the respiratory apparatus due to loss of mitochondrial function and a rapid onset of tissue death (O’Sullivan *et al*., 2013). In the long term (e.g. days to weeks), higher growth temperature causes a change in energy demand and membrane fluidity that results in reduced temperature-normalised *R*_dark_ (i.e. normalised to a standard temperature to allow comparisons of rates under different scenarios), reflecting acclimation of *R*_dark_ to the higher growth temperature. However, limited studies in wheat suggest that CO_2_-based temperature-normalised *R*_dark_ measured during the day (*R*_dark_day_) does not acclimate to night warming. Posch *et al*. (2022) reported no evidence of acclimation of *R*_dark_day_ measured at 25 °C (*R*_dark_day_^25^) in wheat to warm nights, with plants grown under warmer (20 °C and 25 °C) nights relative to cool (15 °C) nights showing similar *R*_dark_day_^25^. By contrast, CO_2_-based *R*_dark_ during the night (*R*_dark_night_) measured at 25 °C (*R*_dark_night_^25^) does acclimate to higher night temperature (Impa *et al*., 2019). However, these studies were based on a narrow genotypic range—two genotypes in Posch *et al*. (2022) and six in Impa *et al*. (2019)—and it remains unclear whether this pattern is consistent across broader wheat germplasm. This highlights the need for further work assessing leaf respiratory responses to night warming across a broader range of wheat cultivars.

Breeding advances and agronomic management have played major roles in wheat yield improvements (Richards *et al*., 2014; Robertson *et al*., 2016). For example, genetic improvement has paralleled enhancement of leaf photosynthesis in China (Tian *et al*., 2011; Zheng *et al*., 2011), Mexico (Fischer *et al*., 2022), and Israel (Blum, 1990). However, it is unclear if enhanced leaf photosynthesis (*A*_net_ measured at a specified temperature; e.g. at 25 °C, *A*_net_^25^) or temperature-normalised lower *R*_dark_ would have been maintained under the progressively warmer climate (and warming nights) that have emerged over the last century. Considering the drag on wheat yield by high night-temperature stress globally, a key challenge to increasing future yields is the need to acquire deeper understanding of photosynthetic and respiratory adjustments in historical and modern cultivars to warm nights and their links with other leaf functional traits, such as leaf nitrogen (N) content (Dusenge *et al*., 2020, 2021; H. Wang *et al*., 2020; Crous *et al*., 2022) and leaf mass per unit area (LMA) (Atkin *et al*., 2006; Sage and Kubien, 2007; Manishimwe *et al*., 2022).

In this study, we examine 10 historic and current Australian wheat cultivars released between 1901 and 2012 (i.e. over a century of breeding) in order to understand their photosynthetic and respiratory adjustments to warm nights. The cultivars were exposed to cool or warm nights coupled with a standard day temperature in controlled-environment facilities to assess leaf photosynthetic traits (*V*_cmax_, *J*_max_, *A*_net_), CO_2_-based *R*_dark_ (during the day, *R*_dark_day_, and at night, *R*_dark_night_), and changes in leaf structure and chemical traits (LMA and leaf N content) under warm nights. Acclimation was assessed on fully expanded leaves that developed under the prevailing warm nights, given that acclimation is more prominent in leaves developed under a prevailing temperature rather than in pre-existing leaves exposed to a temperature shift (Rashid *et al*., 2020; Coast *et al*., 2024). We hypothesised that over the last 111 years breeding indirectly selected for the following changes under warm nights: modern cultivars would exhibit higher *A*_net_^25^ in warm-night-grown plants relative to cool-night controls; increased leaf photosynthetic capacity (*V*_cmax_ and *J*_max_) would underpin higher rates of *A*_net_^25^, and there would be reduced respiratory CO_2_ losses, with lower temperature-normalised rates of *R*_dark_day_ and *R*_dark_night_ in plants exposed to warm nights compared with cool-night controls.

## Materials and methods

The experiment was conducted in controlled-environment facilities (glasshouses and growth chambers) at the University of New England (UNE) in Armidale, Australia (30.48°S, 151.63°E, elevation 1021.5 m above sea level) in 2022.

### Plant materials and growth conditions

A set of 10 wheat (*Triticum aestivum*) genotypes were used (Supplementary Table S1) and included commercial Australian varieties spanning over a century of breeding (1901–2012). Germplasm for the experiment was sourced from the IA Watson Grains Research Centre in Narrabri, NSW, Australia (30.27°S, 149.81°E). Seeds of the cultivars were planted in 4.5 l plastic pots (18 cm diameter, 60 cm height) containing Professional Premium Potting Mix with slow-release fertiliser (J.C. and A.T. Searle Pty. Ltd., Kilcoy, QLD, Australia). The emerged plants were thinned to one per plot, and they were raised in glasshouse bays set to day/night temperatures of 20/12 °C, 70% relative humidity, and a natural photoperiod of 12 h for 4 weeks until the tillering stage (Zadok 20–29) (Zadoks *et al*., 1974). During this period, watering was provided manually, and standard glasshouse plant husbandry was applied. At tillering, when all plants had a fully extended third leaf, they were moved into plant growth chambers (Conviron A1000, Conviron, Winnipeg, Canada) and raised for 3–5 weeks (depending on cultivar developmental rate) through stem elongation (Zadok 30–39) until the booting stage (Zadok 40–49).

Three growth chambers were used, equipped with incandescent and fluorescent lamps and set to a 14 h photoperiod with photosynthetic photon flux density (PPFD) of 800–850 µmol m^−2^ and relative humidity of 60%. The experimental set-up was a randomised block design, consisting of two treatments, each of which was applied to three growth chambers (blocks) that contained two pots each of the 10 cultivars randomly arranged within the chambers (i.e. *n*=6 biological replicate plants). The target treatments were the control temperature conditions of 25/12 °C maximum day/minimum night and the high night-temperature conditions of 25/22° C maximum day/minimum night. The mean night temperatures actually recorded over the course of the experiment were 12.2° C for the control treatment and 21.8° C for the high night temperature treatment, giving a mean night-warming magnitude of 8.8° C. The experiment was conducted in two sequential batches, with all control plants grown first, followed by all the high night-temperature plants. Thus, each batch used the same three growth chambers, thereby ensuring that environmental conditions (light, airflow, humidity, and chamber identity) were consistent across treatments and avoided chamber-specific confounding. Plants were watered daily, and the pots were kept in trays containing ∼1 cm water at the bottom to avoid drought stress. No major pests and diseases were observed during the experiment. The maximum and minimum temperatures were maintained for 8 h each in all the chambers with a transition period of 4 h between the maximum day/minimum night temperatures. Temperature and relative humidity (RH) at canopy level were recorded every 5 min using Tiny-tag Ultra 2 temperature/RH data loggers (Gemini Data Loggers UK) in all growth chambers during both treatments.

### Timing of physiological measurements

All physiological measurements were conducted on recently fully developed flag leaves on main tillers. Development rates differed among the cultivars and in response to warm nights and therefore measurements for each cultivar were conducted when the plants reached comparable developmental stages, and were completed within a one-week period. Specifically, physiological measurements were made when the plants were between ear emergence (Zadoks 50–59) and flowering (Zadoks 60–69). This approach ensured that thermal acclimation responses were evaluated within a narrow ontogenetic window and minimised potential variation arising from shifts in source–sink balance during development.

### Photosynthesis and respiration

Gas exchange measurements were conducted using portable infra-red gas analysers (IRGAs; Li-6400XT, LI-COR Inc., Lincoln, NE, USA). The measurements of net CO_2_ assimilation at 25 °C (*A*_net_^25^) were taken using a 6 cm^2^ leaf chamber and the block temperature set to 25 °C. The CO_2_ concentration was fixed at 400 ppm with a flow rate of 500 μmol s^−1^ and light intensity of 1500 μmol m^−2^ s^−1^ of photosynthetically active radiation (PAR). Dark-adapted *R*_dark_day_ and *R*_dark_night_ were recorded with the same IRGAs. The temperature of the headblock of each chamber was set to 25 °C for *R*_dark_day_ and to 20 °C for *R*_dark_night_, and resulted in mean leaf temperatures across the treatments of 25.5 °C for *A*_net_^25^ and *R*_dark_day_, and 21.3 °C for *R*_dark_night_ (Supplementary Fig. S1). Measurements of *A*_net_^25^ and *R*_dark_day_ (after at least 20 min of dark adaptation) were made during the 8 h period of steady temperature during the day and *R*_dark_night_ was measured during the steady-temperature period during the night. To avoid introducing diurnal bias into parameter estimates, plants were randomly allocated to measurement times within the 8 h period of steady temperature during the day, ensuring that no cultivar was consistently measured at a particular time of day.

Dark-adapted respiration measured during the day (*R*_dark_day_) is not equivalent to non-photorespiratory mitochondrial CO_2_ release (i.e. respiration) in the light (*R*_light_). Leaf respiration in the light is often substantially lower than *R*_dark_ and reflects light-dependent suppression of respiratory CO_2_ release by ∼30% (Kirschbaum and Farquhar, 1987; Krömer, 1995; Wang *et al*., 2001; Ayub *et al*., 2011). However, *R*_light_ can vary from 16–140% of *R*_dark_ (see Way *et al*., 2019, and references therein). Estimating *R*_light_ by the Kok, Laisk, Yin or non-rectangular hyperbolic model methods (Kok, 1956; Laisk, 1977; Fang *et al*., 2022; Yin and Amthor, 2024) relies on additional assumptions and are not directly comparable to dark-adapted measurements. While *R*_light_ plays an important role in day-time carbon balance, our objective here was to examine thermal acclimation of *R*_dark_, and using a single dark-adapted method allowed robust comparisons across cultivars and treatments, and with previous studies. This also avoided conflating biological differences with differences arising from the measurement technique.

### Maximum Rubisco activity and electron transport rate

Two Li-6400XT IRGAs were used to assess photosynthetic capacity of the 10 wheat genotypes. Photosynthetic CO_2_-response curves (*A*/*C*_i_ curves) were generated at constant PAR irradiance of 1500 μmol photons m^−2^ s^−1^ by varying the CO_2_ inside the leaf chambers in the following sequence: 400, 40, 60, 100, 150, 250, 400, 400, 600, 800, 1000, 1200, 1400, and 400 μmol mol^−1^. Relative humidity within the chamber was maintained between 40–70% for all measurements. The maximum carboxylation rate at 25 °C, *V*_cmax_^25^, and the maximum electron transport rate at 1500 μmol m^−2^ s^−1^ PAR at 25 °C, *J*_1500_^25^, were calculated using the leaf biochemical model of photosynthesis (Farquhar *et al*., 1980) with kinetic constants derived for wheat (Silva-Perez *et al*., 2018). Response curves of *A*_net_^25^ to the intercellular CO_2_ concentration (*C*_i_) were measured in the mid-section of the flag leaf when the plants reached Zadoks 40–49. The relationship between *A*_net_^25^ and CO_2_ followed the FvCB model (Farquhar *et al*., 1980; von Caemmerer and Farquhar, 1981) with a simple function for limitation by triose phosphate utilisation (TPU) (Sharkey *et al*., 2007). The approach of Gu and Jerome (2010) was used where all possible carboxylation limitation-state combinations were tested, given the required order of limitation states along the *C*_i_ axis (Rubisco limited < electron transport limited < TPU limited) and the minimum number of data necessary for each limitation state (*n*≥2 when Michaelis–Menten constants for Rubisco catalysis of carboxylation, Kc, and oxygenation reactions, Ko, and the photosynthetic CO_2_ compensation point in the absence of mitochondrial respiration in the light, Γ*, are fixed). Representative *A*/*C*_i_ curves with model fits are presented in Supplementary Fig. S2. The R function optim (https://www.rdocumentation.org/packages/stats/versions/3.6.2/topics/optim) was used to minimise the distribution-wise cost function, and the model with the lowest cost function value was accepted after checking for admissibility and, if necessary, testing for co-limited ‘swinging points’ (Gu and Jerome, 2010). These measurements were taken within a day during the same period as the photosynthesis and respiration measurements.

### Determination of leaf mass per area and nitrogen content

Leaf area (cm^2^) was determined using the same leaves used for gas exchange measurements. The leaves were detached from the plants, placed on a pre-calibrated background, and analysed with the ImageJ software. Leaves were dried at 65 °C for 72 h or until constant dry weight was achieved the leaf dry mass per unit area (LMA, g m^−2^) was aclculated. Then, 1 mg of dry leaf sample was weighed and analysed to determine leaf N (%) using an automatic elemental analyser (Leco TruMac Total Carbon, Nitrogen and Sulfur Macro Determinator, LECO Corporation, Michigan, USA). Using the N content and LMA, we then calculated leaf N per area (N_area_, g m^−2^) and leaf N per dry mass (N_mass_, mg g^−1^).

### Statistical analysis

All data analyses were conducted in R (www.r-project.org). Trait responses to night-temperature treatment, cultivar, and their interaction were examined using ANOVA. Data manipulation, visualisation, and statistical modelling were performed using the dplyr, ggplot2, and gridExtra packages in R. For each trait, means were calculated across cultivars for each treatment and year of release.

Chronological trends in physiological traits (e.g. *A*_net_^25^, *R*_dark_, leaf N content) were assessed using both linear and second-order polynomial regressions. Models were fitted separately to the control and high night-temperature datasets, and model selection was based on comparisons of adjusted *R*^2^ and Akaike Information Criterion (AIC). *F*-tests were also used to determine whether the polynomial models provided statistically significant improvements over the linear models. Non-linear models were adopted only when they satisfied all three criteria: higher adjusted *R*^2^ (by >0.02), lower AIC, and significant *F*-test (*P*<0.05).

To enable comparison of *R*_dark_day_ measured at 25 °C (*R*_dark_day_^25^) and *R*_dark_night_ measured at 20 °C (*R*_dark_night_^20^), we used the global polynomial respiration-temperature model (GPM) of Heskel *et al*. (2016) to estimate *R*_dark_night_ at 25 °C (*R*_dark_night_^25^) for each individual leaf *R*_dark_night_^20^ measurement, as follows:


(1) $$\ln(R)=a+0.1012T\text{–}0.0005T^{2}$$

where ln(*R*) is the log-transformed rate of leaf *R*_dark_night_ at a given leaf temperature *T*, and *a* is a coefficient derived from resolving Equation 1 for *R*_dark_night_^20^ and *T* =20 °C. The mean estimate of *a* was −2.1443 (95% confidence intervals of −2.2620 and −2.0266) compared to Heskel *et al*. (2016) with global mean *a* of −2.2276 (−2.3966 and −2.0586). Differences between *R*_dark_day_^25^ and *R*_dark_night_^25^ were tested using two-sample Welch’s *t*-tests as the measurements were independent observations, samples sizes were unequal, and the variances were not assumed to be equal. Unless otherwise stated, results presented for *R*_dark_night_ are of *R*_dark_night_^20^.

To evaluate thermal acclimation across breeding years, derived trait ratios (e.g. *J*_1500_^25^/*V*_cmax_^25^, *R*_dark_day_/*V*_cmax_^25^, and *R*_dark_night_/*V*_cmax_^25^) were calculated and modelled similarly. Where relevant, trait relationships with leaf N were also explored using regression analysis to assess the relationship between biochemical and physiological traits under control and high night-temperature conditions.

## Results

### No systematic change in net photosynthesis in over a century of wheat breeding except in terms of leaf N

**Fig. 1.**
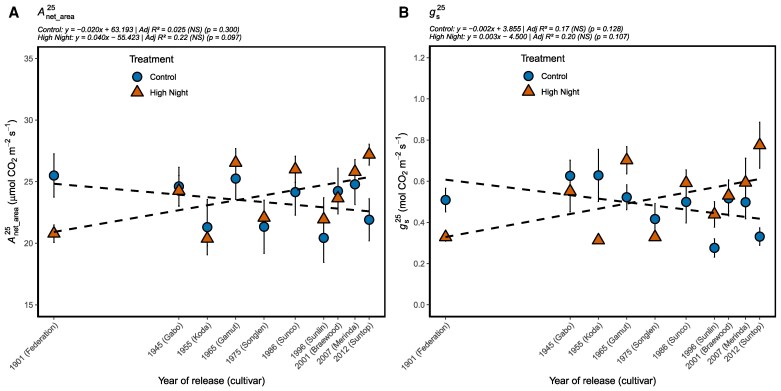
Effects of high night temperature on photosynthesis and stomatal conductance in 10 Australian wheat cultivars released between 1901 and 2012. Plants were treated with day/night temperatures of 25/12 °C (control) or 25/22 °C (high night). (A) Net rates of photosynthetic CO_2_ assimilation per unit leaf area measured at 25 °C (*A*_net_area_^25^) and (B) stomatal conductance measured at 25 °C (*g*_s_^25^). Data are means (±SEM), *n*=2–6. Regression equations and adjusted *R*^2^ values are shown; NS, not significant. Where the regression slope is not significantly different from zero this is indicated by black dashed lines and NS next to the corresponding adjusted *R*^2^.

Rates of *A*_net_^25^ per unit leaf area differed among the 10 cultivars across both control and high night-temperature treatments, and a significant treatment effect was observed (*F*_3,114_=3.05, *P*=0.031; Fig. 1A). While the main effect of year of release was not significant when fitted as a continuous variable (*P*=0.180), a significant interaction (*P*=0.006) indicated that the cultivar rankings differed between the treatments. For instance, the early cultivar Federation (1901) exhibited the highest *A*_net_^25^ under control conditions (25.5 µmol CO_2_ m^−2^ s^−1^) but showed a pronounced decline (19%) under high night temperature. By contrast, the modern cultivar Suntop (2012), which had a modest *A*_net_^25^ under control conditions showed a significant increase under the high night temperature (24%). Overall, there was no significant trend as a function of year of release under either treatment, suggesting that breeding over time has not led to a systematic change in *A*_net_^25^. The associated stomatal conductance of the cultivars measured at 25 °C and their responses to night temperature were similar to those recorded for *A*_net_^25^ (Fig. 1B). The slopes of the *A*_net_^25^ versus year of release did not differ between the two treatments (despite being slightly negative under control and positive under high night temperature), and were not significantly different from zero (*P*>0.05). Similarly, no significant trends were observed for *A*_net_^25^ versus year of release when expressed per unit dry mass (*A*_net_DM_) or fresh mass (*A*_net_FM_; Supplementary Fig. S3A, B). However, when expressed as per unit nitrogen (*A*_net_N_; Supplementary Fig. S3C) a significant decline was observed under control conditions, indicating a potential decoupling of photosynthesis from leaf N content in modern cultivars under non-warming night conditions.

### Photosynthetic capacity in modern cultivars is reduced under warm nights but triose phosphate utilization is stable with year of release

**Fig. 2.**
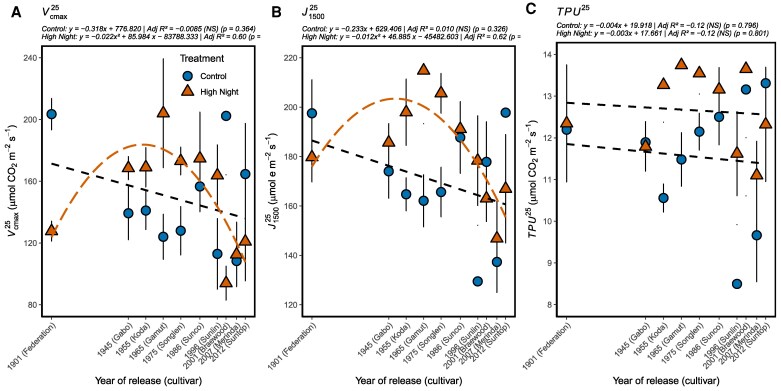
Effects of high night temperature on photosynthetic efficiency traits in 10 Australian wheat cultivars released between 1901 and 2012. Plants were treated with day/night temperatures of 25/12 °C (control) or 25/22 °C (high night), and all measurements were made at 25 °C. (A) Maximum rate of Rubisco carboxylation (*V*_cmax_), (B) rate of electron transport (*J*_1500_), and (C) rate of triphosphate utilisation (TPU). Data are means (±SEM), *n*=2–6. Regression equations and adjusted *R*^2^ values are shown; NS, not significant. Non-significant relationships are shown as black dashed lines, whilst significant relationships are colour-coded according to the key.

To further examine the mechanisms underlying the effects on *A*_net_^25^, *V*_cmax_, *J*_1500_, and TPU were determined from *A*/*C*_i_ curves at 25 °C (*V*_cmax_^25^, *J*_1500_^25^, and TPU^25^, respectively). For each measured leaf, unreliable estimates of *V*_cmax_^25^, *J*_1500_^25^, and TPU^25^ with *A*_net_^25^ versus chloroplastic CO_2_ (*A*_net_/*C*_c_) curves showing obvious errors were omitted from further analyses of photosynthetic capacity (see Supplementary Fig. S2 for examples). Under control conditions, *V*_cmax_^25^ showed no significant trend with year of release, even though it varied significantly among cultivars (Fig. 2A). For example, the mean *V*_cmax_^25^ of Federation (1901) at 204 µmol CO_2_ m^2^ s^−1^ was almost double that of Merinda (2007) at 108 µmol CO_2_ m^2^ s^−1^. By contrast, under high night temperature a significant relationship was observed with a peak around 1955 and a subsequent sharp decline, indicating reduced carboxylation capacity in more recent cultivars when exposed to warm nights.

**Fig. 3.**
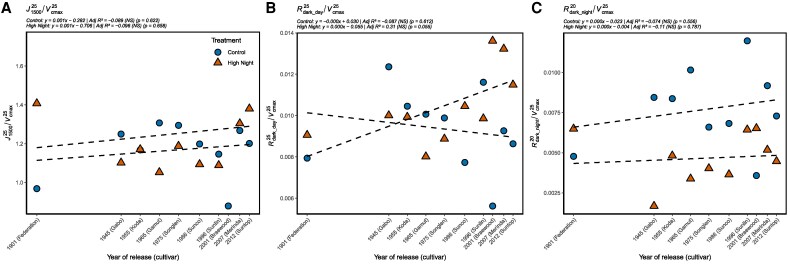
Effects of high night temperature on relative rates of photosynthetic efficiency and dark respiration in 10 Australian wheat cultivars released between 1901 and 2012. Plants were treated with day/night temperatures of 25/12 °C (control) or 25/22 °C (high night). (A) Ratio of the rate of electron transport measured at 25 °C (*J*_1500_^25^) to the rate of Rubisco carboxylation measured at 25 °C (*V*_cmax_^25^). (B) Ratio of leaf dark respiration during the day measured at 25 °C (*R*_dark_day_^25^) to *V*_cmax_^25^ and (C) ratio of leaf dark respiration at night measured at 20 °C (*R*_dark_night_^20^) to *V*_cmax_^25^. Data are means (±SEM), *n*=2–3. Regression equations and adjusted *R*^2^ values are shown; NS, not significant.

Only two cultivars showed significant down-regulation of *V*_cmax_^25^ under warm nights compared with the control, namely Federation (1901) and Braewood (2001). Of the other eight, five mostly older cultivars (1945–1996) showed increased *V*_cmax_^25^ under warm nights and three (including two new cultivars) showed no significant difference. The greatest increase was exhibited by Gamut (1965). *J*_1500_^25^ largely followed the same patterns as *V*_cmax_^25^ under control and high night temperatures, with no significant trend detected under control conditions and a fitted peak for year of release around 1955 (Fig. 2B). TPU^25^ exhibited little variation under either treatment, with the mean value under both control and high night temperatures being 12.5 µmol CO_2_ m^2^ s^−1^ and with no difference in the slopes (Fig. 2C), suggesting stability in TPU^25^ capacity across breeding history. Under warm night conditions, most older cultivars had significantly higher *V*_cmax_^25^ and *J*_1500_^25^ values than newer cultivars (Fig. 2A, B). The difference was largest forGamut (1965) for both parameters, and consequently the *J*_1500_^25^/*V*_cmax_^25^ ratio was highest for this cultivar (Fig. 3A).

### Warm nights increase leaf respiration during the day but decrease it during the night

**Fig. 4.**
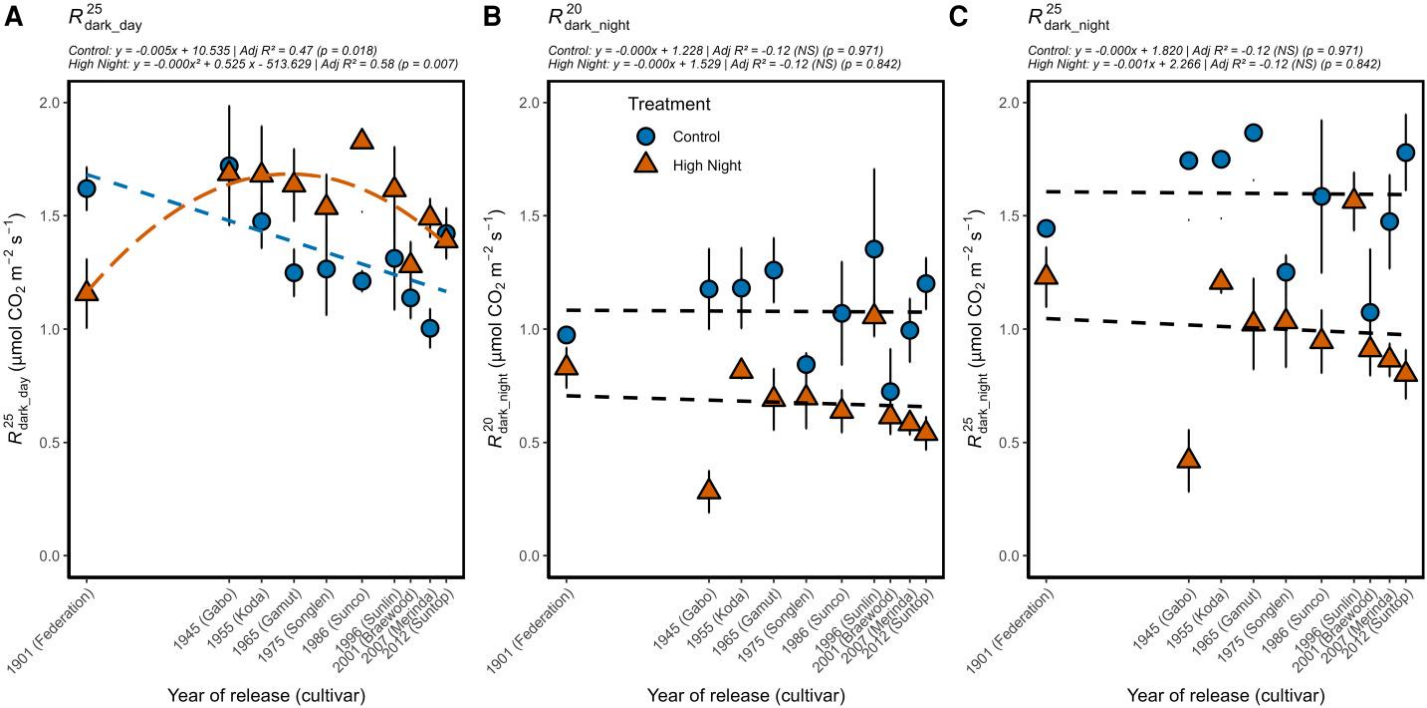
Effects of high night temperature on leaf CO_2_-based respiration in 10 Australian wheat cultivars released between 1901 and 2012. Plants were treated with day/night temperatures of 25/12 °C (control) or 25/22 °C (high night). Leaf dark respiration was measured (A) during the day at 25 °C (*R*_dark_day_^25^) and (B) during the night at 20 °C (*R*_dark_night_^20^). To allow a direct comparison with *R*_dark_day_^25^, (C) the temperature-normalised dark respiration during the night at 25 °C (*R*_dark_night_^25^) was estimated using the global polynomial respiration-temperature model of Heskel *et al* (2016). Data are means (±SEM), *n*=2–6. Regression equations and adjusted *R*^2^ values are shown; NS, not significant. Non-significant relationships are shown as black dashed lines, whilst significant relationships are colour-coded according to the key.

Leaf dark respiration measured during the day at 25 °C (*R*_dark_day_^25^) and during the night at 20 °C (*R*_dark_night_^20^) showed different trends between the control and warm night treatments and varied among cultivars. *R*_dark_day_^25^ exhibited a significant linear decline with year of release under control conditions (Fig. 4A and it also declined under warm nights, but only from 1945 with the release of Gabo. The curvilinear response of *R*_dark_day_^25^ under high night temperature was largely driven by the oldest cultivar Federation (1901), which had the lowest value among all the genotypes. Overall, values of *R*_dark_day_^25^ were either stable or increased under warm nights except for Federation (1901), which had a lower value in plants treated to warm nights. By contrast, *R*_dark_night_^20^ showed no discernible trend with year of release under either control or warm night conditions, and for most cultivars it was significantly reduced under the high night temperature (Fig. 4B), suggesting that *R*_dark_night_ thermally acclimated in most cases. At the common temperature of 25 °C, temperature-normalised *R*_dark_night_^25^ was higher than *R*_dark_day_^25^ under control conditions, but the reverse was the case under high night conditions (Supplementary Fig. S4; also compare Fig. 4A versus 4C). Warm nights resulted in the relationship between the *R*_dark_day_^25^/*V*_cmax_^25^ ratio and tear of release being changed from a negative (control) to positive (warm night) slope (Fig. 3B). Additionally, warm nights markedly lowered the *R*_dark_night_^20^/*V*_cmax_^25^ ratios compared with the controls (Fig. 3C).

### Leaf nitrogen content increases but leaf mass per area remains unchanged under warm nights

**Fig. 5.**
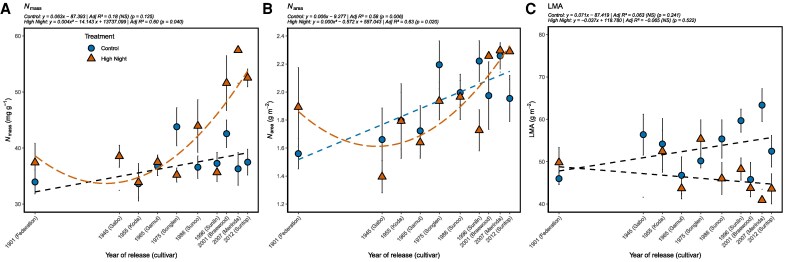
Effects of high night temperature on leaf functional traits in 10 Australian wheat cultivars released between 1901 and 2012. Plants were treated with day/night temperatures of 25/12 °C (control) or 25/22 °C (high night). (A) Nitrogen per unit leaf mass, *N*_mass_, (B) nitrogen per unit leaf area, *N*_area_, and (C) leaf mass per unit area (LMA). Data are means (±SEM), *n*=2–6. Regression equations and adjusted *R*^2^ values are shown.

**Fig. 6.**
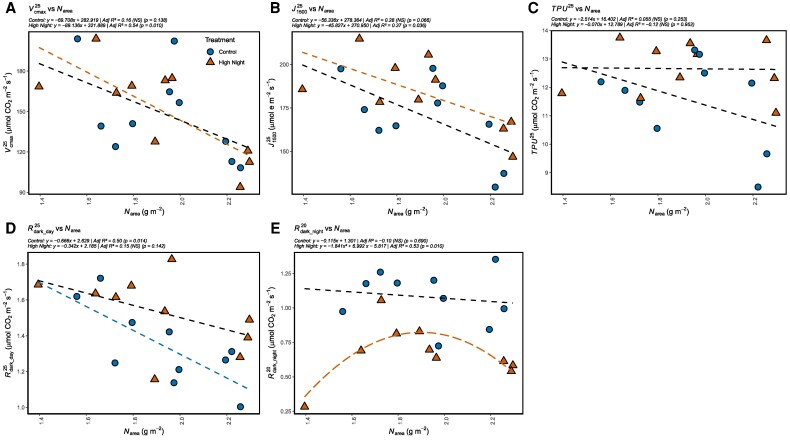
Effects of high night temperature on relationships between leaf photosynthetic capacity and nitrogen per unit leaf area (N_area_) in 10 Australian wheat cultivars released between 1901 and 2012. Plants were treated with day/night temperatures of 25/12 °C (control) or 25/22 °C (high night). (A) Maximum rate of Rubisco carboxylation at 25 °C (*V*_cmax_^25^) (B) rate of electron transport at 25 °C (*J*_1500_^25^), (C) rate of triphosphate utilisation at 25 °C (TPU^25^), (D) leaf dark respiration during the day at 25 °C (*R*_dark_day_^25^), and (E) leaf dark respiration at night at 20 °C (*R*_dark_night_^20^). Data are means (±SEM), *n*=2–6. Regression equations and adjusted *R*^2^ values are shown.

For both treatments, leaf N content on a per mass and per area basis (N_mass_ and N_area_) increased with year of release (i.e. was different among cultivars), and this was more marked in response to warm nights (Fig. 5A, B). By contrast, leaf mass per unit area (LMA) showed no consistent temporal trend under either treatment (Fig. 5C). Leaf N_area_ was associated linearly with the photosynthetic capacity traits *V*_cmax_^25^ and *J*_1500_^25^ under both control and high night temperature (Fig. 6A, B). The relationship was strongest for *V*_cmax_^25^ (adjusted *R*^2^ = 0.54) under warm nights followed by *J*_1500_^25^ (adjusted *R*^2^ = 0.37), also under warm nights. Irrespective of leaf N_area_  *J*_1500_^25^ was higher under warm nights than control conditions. Leaf TPU^25^ appeared to decrease with N_area_ under control condition but this trend was not significant. Under high night condition TPU was insensitive to increasing N_area_ (Fig. 6C). Leaf *R*_dark_day_^25^ declined with increasing N_area_ under control but not under warm nights, while *R*_dark_night_^20^ exhibited a non-linear response to N_area_ under warm nights (Fig. 6D, E).

## Discussion

Global wheat yields have increased over the last several decades due to advances in breeding and improved agronomic management (Fischer *et al*., 2014; Richards *et al*., 2014). It is assumed that conventional breeding and selection for yield increases has inadvertently selected for enhanced photosynthesis (*A*_net_) or lower *R*_dark_. However, with rising night temperatures causing a drag on wheat yield, it is unclear how well these physiological traits acclimate to warm nights. In our study, leaf *A*_net_ measured at 25 °C (*A*_net_^25^) showed no systematic change with year of release for 10 selected Australian wheat cultivars spanning over a century (1901–2012) of breeding, except when expressed per unit N, where older cultivars exhibited greater *A*_net_^25^ under warm nights (Supplementary Fig. S3C). In terms of the biochemical components that underpin C_3_ photosynthetic capacity, modern cultivars exhibited reduced *V*_cmax_^25^ and *J*_1500_^25^ under warmer nights compared with the control temperature (Fig. 2), contrary to the hypothesis that conventional breeding has inadvertently selected for enhanced photosynthesis. Leaf *R*_dark_ measured during the day at 25 °C (*R*_dark_day_^25^) and during the night at 20 °C (*R*_dark_night_^20^) or estimated at 25 °C (*R*_dark_night_^25^) showed consistently different responses to warming: leaf *R*_dark_day_^25^ declined with year of release under control conditions but was stimulated under warm nights, whereas leaf *R*_dark_night_^20^ and *R*_dark_night_^25^ remained unchanged across time but were significantly lower under warm nights (Fig. 4). These contrasting thermal sensitivities suggest differential metabolic control of *R*_dark_day_ and *R*_dark_night_, possibly reflecting circadian regulation, substrate availability, or partitioning of energy demands during light and dark periods. Understanding the mechanisms driving these divergent responses offers a pathway to inform targeted breeding strategies for wheat cultivars that are better adapted to future climates.

### Limited gains have been made in photosynthetic rate, but a stable response to warm nights might enhance yield resilience

Historical yield gains in Australia do not appear to have been underpinned by systematic improvements in leaf photosynthesis across the cultivars released between 1901 to 2012 according to our results under control night temperatures. This suggests that breeding strategies focused on yield and stress resilience have not inadvertently selected for increased photosynthetic rates or capacity. Nonetheless, enhancing photosynthesis remains a viable strategy for boosting wheat productivity under future climates (Yin *et al*., 2022; Burgess *et al*., 2023; Gracia-Romero *et al*., 2023; Croce *et al*., 2024), especially by maximising the efficiency of converting intercepted radiation into biomass (Garcia *et al*., 2023). Several physiological strategies have been proposed to improve photosynthetic efficiency, including the following: increasing the catalytic performance of Rubisco (ribulose 1,5 biphosphate carboxylase/oxygenase) and enhancing electron transport and mesophyll conductance (Parry *et al*., 2011; Whitney *et al*., 2011; Prins *et al*., 2016; Wu *et al*., 2019; Yin *et al*., 2022); exploiting photosynthetic contributions of non-foliar tissues including spikes, tillers, and awns (Simkin *et al*., 2020; Vicente *et al*., 2024); modifying canopy architecture to improve light interception and increase radiation use efficiency (Guarin *et al*., 2022); and engineering photorespiratory pathways to reduce CO_2_ losses (Hagemann and Bauwe, 2016; South *et al*., 2018; Garcia *et al*., 2023). Modelling estimates suggest that simultaneous improvements in Rubisco activity, electron transport, and mesophyll conductance could increase wheat yield by 5.3–12.1% (Wu *et al*., 2019). However, other studies caution that gains in photosynthesis are unlikely to translate to higher yields unless coupled with coordinated improvements in N acquisition, assimilation, and remobilisation to grains (Sinclair *et al*., 2019, 2023; De Souza *et al*., 2023).

Acclimation of photosynthesis to warming typically involves enhancement of *A*_net_ at a common temperature in warmer-grown plants and an increase in the optimum temperature (*T*_opt_) of *A*_net_ at the warmer growth temperature (Berry and Bjorkman, 1980; Way and Yamori, 2014). In our study, the stable *A*_net_^25^ under warming across most cultivars (Fig. 1A, B) could be due to limited sensitivity to night temperature in the range 20–25 °C. Our findings are consistent with previous studies in wheat; for example, Coast *et al*. (2024) observed *A*_net_^25^ stability or increases under nights of 20 °C and 25 °C in three out of four genotypes, and Impa *et al*. (2019) reported stable *A*_net_^26^ in five of six winter wheat genotypes under day/night temperature treatments of 26/23 °C. In our present study, where the mean night-warming magnitude was 8.7 °C, *A*_net_^25^ of nine of the 10 cultivars was not negatively affected by warm nights. But the nine cultivars also did not show typical acclimation of photosynthesis to higher temperature, namely constructive acclimation, which is described by Way and Yamori (2014) as both an increase in *A*_net_^25^ and its *T*_opt_ at the warmer growth temperature. The exception was the earliest released cultivar, Federation (1901), which consistently exhibited a decline in *A*_net_^25^ under warming (i.e. detractive acclimation), regardless of whether it was expressed per unit area, mass, or nitrogen. Previous studies have shown that historical yield gains in wheat have not typically been accompanied by steady improvements (typical acclimation) in leaf photosynthetic (Long *et al*., 2006; Parry *et al*., 2011; Driever *et al*., 2014; Fischer *et al*., 2014; Garcia *et al*., 2023), although recent more evidence indicates that light-saturated photosynthesis during seed-fill has increased over breeding history (Ding *et al*., 2025). Our results are in agreement with a recent study by Coast *et al*. (2024), showing that modern wheat cultivars maintain *A*_net_^25^ under warm-night conditions. Given that rising night temperature is a hallmark of climate change (IPCC, 2013), the ability of wheat leaves to at least maintain photosynthetic performance under warm nights might represent an important physiological mechanism contributing to yield stability in future climates.

### Photosynthetic capacity has decreased in modern cultivars; however different cultivar sensitivities to warm nights provide opportunities for increasing photosynthesis

Under saturating light and adequate water supply, photosynthesis in C_3_ species such as wheat is limited by the kinetics of carbon fixation (*V*_cmax_) and RuBP regeneration via the maximum electron transport rate (*J*_max_) (Farquhar *et al*., 1980; Farquhar and Sharkey, 1982). These two processes underpin photosynthetic capacity and are sensitive to thermal and genotypic variation. In our study, photosynthetic capacity showed no clear trend with cultivar year of release under control night temperatures (Fig. 2), consistent with a study of historical soybean germplasm (Koester *et al*., 2016). However, under warm nights, the photosynthetic capacity declined in more recent cultivars, and most exhibited higher *V*_cmax_^25^, *J*_1500_^25^, and TPU^25^ under warming. The exception was Federation, released in 1901, and Suntop, released in 2012, which showed higher *V*_cmax_^25^ and *J*_1500_^25^ under control conditions. These results show that there are modern cultivars able to thermally acclimate to warm nights and thus maintain or improve photosynthetic capacity under such conditions. Although the cooler-night preference of these modern cultivars might align with a hypothesis of greater N allocation to photosynthetic machinery at lower temperatures, their absolute *V*_cmax_^25^ and *J*_1500_^25^ values were lower than those of historical cultivars. Importantly, these reductions were not associated with decreased leaf N, which increased with cultivar year of release and warm night treatment (Fig. 5A, B). This implies a possible shift or lower proportional allocation of N to photosynthetic proteins such as Rubisco or components of the electron transport chain in modern cultivars. These results are consistent with earlier reports that warming reduces *V*_cmax_ through changes in protein allocation rather than total N (Scafaro *et al*., 2017; Crous *et al*., 2018; Dusenge *et al*., 2020), and they align with recent meta-analyses showing reduced *V*_cmax_^25^ in warm-grown plants (H. Wang *et al*., 2020; Crous *et al*., 2022). Our results also support the idea that selection for yield has not coincided with increased photosynthetic capacity, and might have inadvertently favoured lines with lower biochemical capacities (Driever *et al*., 2014) under warming. However, it is likely that modern wheat cultivars can maintain *A*_net_^25^ at higher night temperatures even with less protein invested in the photosynthetic machinery, because they have more efficient photosynthetic processes. This could be beneficial for reducing *R*_dark_ (in particular *R*_dark_night_), which would translate to less maintenance respiration required for expensive Rubisco protein turnover and synthesis.

The differing thermal sensitivities of *V*_cmax_^25^ and *J*_1500_^25^ (Fig. 2) suggest underlying biochemical divergence. Electron transport processes are more dependent on thylakoid membrane structure and redox balance, rendering *J*_1500_^25^ more vulnerable to high temperatures (Yamori *et al*., 2014). Rubisco carboxylation, while also temperature-sensitive, might be comparatively more buffered due to its enzymatic activation properties. The distinct trajectories of *V*_cmax_^25^ and *J*_1500_^25^ under warm nights further manifested in increased *J*_1500_^25^/*V*_cmax_^25^ ratios in modern cultivars (Fig. 3A), suggesting a decoupling of RuBP regeneration and carboxylation under warming. By contrast, TPU^25^ remained stable across treatments and genotypes (Fig. 2C), suggesting that it was not limiting under warm night stress. This is consistent with previous studies that have reported TPU stability under moderate stress (H. Wang *et al*., 2020), and highlights the early steps of the Calvin cycle as key targets for improving photosynthetic resilience to warming.

### Wheat leaf *R*_dark_night_, but not *R*_dark_day_, acclimates to moderate warm nights

A widely proposed theory for yield loss due to warm nights has been stimulation of dark respiratory CO_2_ release, which might be coupled with reduced photosynthetic capacity. In response to sustained increases in temperature, plants acclimate by reducing their respiration rate when measured at a set temperature (Atkin and Tjoelker, 2003). In our study, warm nights consistently reduced *R*_dark_night_ while stimulating *R*_dark_day_ (Fig. 4), indicating that nocturnal respiration acclimated to warming whereas daytime respiration did not. These divergent responses are consistent with previous observations in wheat showing *R*_dark_night_ acclimation to night warming (Impa *et al*., 2019), and no acclimation of *R*_dark_day_^25^ under high night temperature regimes (Posch *et al*., 2022). In the latter study, the only decrease in *R*_dark_day_ that occurred was under an extreme night temperature (25 °C) and a minimal diurnal range (26/25 °C), suggesting that severe treatments might drive atypical responses; however, the decrease was not significant (i.e. no acclimation). In our present study, the divergent responses of *R*_dark_day_^25^ and temperature-normalised *R*_dark_night_^25^ to nocturnal warming resulted in an overall reduction in respiratory CO_2_ release (∼12%), as the stimulation of *R*_dark_day_ was insufficient to offset the reduction of *R*_dark_night_: *R*_dark_day_^25^ would have required a 31% increase to compensate fully.

Our study agrees with earlier reports of acclimation of *R*_dark_night_ to night warming observed in other plants/crops treated at moderate night temperatures (low- to mid-20 °C) including rice (Peraudeau *et al*., 2015), soyabean (Djanaguiraman *et al*., 2013), loblolly pine (*Pinus taeda*; Will, 2000), and *Stipa krylovii* (Chi *et al*., 2013). The latter study also showed that leaves of *S. krylovii* did not acclimate *R*_dark_day_ to nocturnal warming. However, our results contradict those of increased *R*_dark_night_ to warm nights (i.e. limited or no acclimation of temperature-normalised rates of *R*_dark_night_; Fig. 4C) reported by others for cotton (Loka and Oosterhuis, 2010), maize (Kettler *et al*., 2022), sorghum and sunflower (Manunta and Kirkham, 1996), rice (Bahuguna *et al*., 2017), and soyabean (Djanaguiraman *et al*., 2013). The differences in acclimation responses might be due to imposition of very high night temperatures (>25 °C) or little diurnal amplitude (e.g. 26/25 °C day/night regime) in previous studies. We note that protocols for assessing acclimation of *R*_dark_ differ among studies: some measure *R*_dark_ at the growth temperatures of each treatment (e.g. Manunta and Kirkham, 1996; Djanaguiraman *et al*., 2013; Bahuguna *et al*., 2017; Impa *et al*., 2019), whereas others quantify its acclimation at a common reference temperature, following the framework of Loveys *et al*. (2003) and Atkin and Tjoelker (2003) (Chi *et al*., 2013; Peraudeau *et al*., 2015; Reich *et al*., 2016; Huntingford *et al*., 2017; Coast *et al*., 2021). Our approach followed the latter.

The uncoupling of *R*_dark_night_ and *R*_dark_day_ from night temperature suggests that their acclimation might be regulated independently. This probably reflects diurnal variation in leaf metabolic status, demand for respiratory products, and the source of substrate supply used in the mitochondria for respiration. At night, sucrose is derived from circadian-controlled synthesis of remobilized transitory starch degraded in the chloroplast (Smith and Stitt, 2007; Smith and Zeeman, 2020), whereas in the day it is synthesized from triose phosphates produced by the Calvin–Benson–Bassham cycle (Gessler *et al*., 2009, 2017). These implies that *R*_dark_day_, constrained by its coupling with photosynthesis, might be less free to down-regulate under nocturnal warming.

Respiratory acclimation has been linked to foliar N, reflecting the leaf N–respiratory scaling relationship and the dependence of respiration on N-rich enzymes and co-factors that make up a large proportion of total N in leaves (Tjoelker *et al*., 1999; Atkin and Tjoelker, 2003; Atkin *et al*., 2015). In our study, leaf N scaled curvilinearly with *R*_dark_night_ under warm nights but weakly and negatively with *R*_dark_day_ under warm nightse (Fig. 6D, E), further highlighting their divergent responses. The coupling of leaf N and *R*_dark_night_ under warm nights suggest enzyme limitation of respiratory capacity (Ryan *et al*., 1996) and highlights the potential use of leaf N as a predictor of *R*_dark_night_ in crop and Earth system models (Fisher *et al*., 2014; Atkin *et al*., 2015). However, leaf N accounted for only about half of the variation in *R*_dark_night_ responses, implying that other factors also contribute, such as substrate availability (Covey-Crump *et al*., 2002), changes in soluble carbohydrate concentrations (Atkin *et al*., 2000b), altered enzyme capacity (Atkin and Tjoelker, 2003), and differences in the temperature sensitivity of enzymatic steps regulating day versus night respiration.

Alternatively, given that *V_c_*_max_ controls the supply of respiratory substrates (O’Leary *et al*., 2019) and that respiratory ATP is required to maintain protein turnover in the photosynthetic apparatus (Dusenge *et al*., 2021), acclimation of *R*_dark_night_ might reflect a strategy to sustain optimal photosynthetic capacity (H. Wang *et al*., 2020; Sturchio *et al*., 2022). This interpretation is consistent with our finding that decreased *R*_dark_night_ coupled with reduced but more efficient *V*_cmax_ and *J*_max_, especially in modern cultivars, resulted in maintained *A*_net_^25^. However, these patterns are not consistent with typical photosynthetic acclimation (Way and Sage, 2008; Way and Yamori, 2014), emphasising the need for further work to disentangle the regulatory links between *R*_dark_day_ and photosynthetic capacity. Whether *R*_dark_night_ acclimation in wheat represents Type I or Type II acclimation (Atkin and Tjoelker, 2003) was not examined in our study and remains to be tested, but our findings indicate that *R*_dark_night_ exhibits greater plasticity and thermal sensitivity than previously recognised.

By contrast, the lack of *R*_dark_day_ acclimation is probably linked to its coordination with photosynthetic capacity. Under warm nights, the ratio *R*_dark_day_/*V*_cmax_ increased with year of release (Fig. 3A), suggesting that higher daytime respiratory demand might have depleted soluble carbohydrate pools, thereby constraining catalytic activity and limiting thermal downregulation of *R*_dark_day_.

### Towards improved parameterisation of respiration in crop and Earth system models

Our finding that *R*_dark_night_ acclimates to moderate warm nights whereas *R*_dark_day_ does not, has important implications for crop and Earth system models and the reliable estimation of terrestrial carbon emissions. Most models assume that measurement of *R*_dark_day_ (after 30 min of dark adaptation during the day to avoid the post-illumination photorespiratory CO_2_ burst and light-enhanced dark respiration) equates to *R*_dark_night_ (Atkin *et al*., 2000a; Padmasree *et al*., 2002; Huntingford *et al*., 2017; Butler *et al*., 2021), thus conflating *R*_dark_day_ and *R*_dark_night_ responses. While *R*_dark_day_ can be similar to *R*_dark_night_ in some situations (Florez-Sarasa *et al*., 2012), there is growing evidence that the two differ (Gessler *et al*., 2009, 2017; Fan *et al,* 2024; Zheng *et al*., 2025). As such, even though both are assessed under dark conditions, their thermal sensitivities are probably different (Bruhn *et al*., 2022, 2024a; Zheng *et al*., 2024). Our results therefore highlight that models reliant on *R*_dark_day_ measured in darkness as a proxy for *R*_dark_night_ measured in darkness would lead to overestimation of night-warming-induced respiratory carbon losses over long periods. This emphasises the need for a more complete understanding of the degree to which *R*_dark_night_ and *R*_dark_day_ responses to warm nights differ within and among plant species. Improving parameterisation of Earth system models will require dynamic temperature-response functions that explicitly distinguish between *R*_dark_day_ and *R*_dark_night_ and incorporate their capacity for acclimation. We note that in our study *R*_dark_day_ is a dark-adapted measure obtained during daylight hours and reflects intrinsic *R*_dark_; it does not quantify realised daytime respiration in the light, which is typically lower due to light inhibition.

### Future research suggestions

Research is required to identify quantitative trait loci associated with *R*_dark_night_, *V*_cmax_, and *J*_max_ under warm night conditions. This will be critical for uncovering the genetic basis of acclimation and enabling marker-assisted selection. Progress in breeding wheat cultivars with enhanced tolerance to warm nights can be supported by understanding the genetic architecture underlying respiratory and photosynthetic traits, particularly *R*_dark_night_. While respiration is a complex/polygenic trait influenced by many loci with small effects and strongly modulated by environmental conditions, recent studies suggest that it is nonetheless genetically tractable. For example, wheat leaf respiration has been shown to be under both genetic and environmental control (Gaju *et al*., 2025), and genome-wide association studies are beginning to reveal the underlying complexity of respiration in wheat and other crops (Guo *et al*., 2021; Bulut *et al*., 2023).

Research is also required to characterise the type and drivers of *R*_dark_night_ acclimation to warm nights. Attention should also be given to improving the quantification and representation of *R*_dark_night_ temperature responses in crop and Earth system models (Bruhn *et al*., 2022, 2024b, 2025), and to ascertaining whether the thermal sensitivities of *R*_dark_night_ CO_2_ efflux and O_2_ uptake are coordinated or decoupled (Coast *et al*., 2021; Bruhn *et al*., 2024a). Simultaneous measurements of both these *R*_dark_night_ respiratory fluxes would allow estimation of the respiratory quotient (RQ, the ratio of moles of CO_2_ released to moles of O_2_ consumed) and indicate the substrate being oxidised for mitochondrial respiration. Changes in RQ can reveal patterns of substrate use and shifts in respiratory metabolism under high night temperature stress. Future research could also focus on understanding whether the pattern seen for *V*_cmax_^25^ (i.e. decreasing under warm nights in modern cultivars but probably more efficient) is held more widely. This might be related to the abundance of and activation state of Rubisco. Also to be explored is why *A*_net_ is insensitive to moderate night temperatures in contrast to *V*_cmax_ and *J*_max_.

These research directions are relevant not only to wheat but also to other economically important cereal crops that are highly sensitive to night warming, including rice, barley, and sorghum (Peng *et al*., 2004; Prasad and Djanaguiraman, 2011; García *et al*, 2015, 2016). The trait-based insights from our study provide a physiological foundation for breeding crops with improved resilience to nocturnal warming. By targeting leaf gas-exchange traits, in particularly *R*_dark_night_, future breeding efforts could enhance energy use efficiency and yield stability under climate change.

## Conclusion

Our study has shown that wheat breeding over the past century in Australia, which has focused on selection for higher yields, has inadvertently maintained net CO_2_ assimilation at 25 °C despite declines in *V*_cmax_ and *J*_1500_ under warm night conditions. Warm nights induced divergent responses in leaf respiration: while *R*_dark_day_ at 25 °C was stimulated, temperature-normalised *R*_dark_night_ acclimated (i.e. temperature-normalised rates were lower under warm nights), exhibiting a down-regulation of respiratory CO_2_ release. The reduction in *R*_dark_night_ is likely driven by reduced demand for maintenance respiration, potentially reflecting lower investment in Rubisco protein turnover and synthesis. The capacity for wheat *R*_dark_night_ acclimation to warm nights has been largely overlooked, despite its potential importance in reducing respiratory carbon loss to the atmosphere and mitigating yield declines under climate change.

## Supplementary Material

erag106_Supplementary_Data

## Data Availability

The data that support the findings of this study and the R codes for visualisation of results are openly available at Dryad Digital Repository, https://doi.org/10.5061/dryad.jq2bvq8ph (Rana Shahi *et al*., 2026).

## Supplementary data

The following supplementary data are available at *JXB* online.

Table S1. Year of release and basic information for the 10 wheat cultivars used in the study.

Fig. S1. Leaf temperatures during measurements of net CO_2_ assimilation, daytime leaf respiration and night leaf respiration under control and high night temperatures.

Fig. S2. Examples of good and bad plots of net rates of photosynthetic CO_2_ assimilation at different intercellular CO_2_ concentration.

Fig. S3. Net rates of photosynthetic CO_2_ assimilation of the 10 cultivars in terms of leaf dry mass, fresh mass, and N content plotted against year of release.

Fig. S4. Rates of leaf *R*_dark_night_ and *R*_dark_day_ at 25 °C either measured during the day at 25 °C or estimated from rates measured during the night at 20 °C.
