# Supplementary material for: Wheat leaf dark respiration acclimates more strongly at night than in the day when responding to nocturnal warming
Source: J Exp Bot. 2026 Feb 26;77(12):3777–92. doi: 10.1093/jxb/erag106 (PMC13293083; doi:10.1093/jxb/erag106)
Supplement: erag106_Supplementary_Data [file erag106_supplementary_data.pdf]

Supplementary Table S1. Pedigrees, year of introduction, and susceptibility traits of the wheat cultivars used in this study.

	Year released	Variety	Notes	References[1]
1	1901	Federation	AH (Australian Hard), Bred by William Farrer, first Australian variety with both rust and drought resistance. Cold stress tolerant	Paull et al. (1998)
2	1945	Gabo	First variety released by University of Sydney, widely planted, Early maturing, short to medium height straw, susceptible to flat smut and rust, successful grown in most districts.	Reeves (1963)
3	1955	Koda	APH (Australian Prime Hard), grown on heavy land for short season	Parish & Stone, (1965) and Reeves (1963)
4	1965	Gamut	ASW (Australian Standard White), spring wheat, rust resistant,	Snape et al. (1979),
5	1975	Songlen	Australian spring wheat with white grain colour	Mares (1983), Wellings et al. (2005) and Snape et al. (1979)
6	1986	Sunco	AH. Moderately resistant to rust. Rot and sprouting resistance. Commercial variety.	Salman et al. (2009)
7	1996	Sunlin	AH, excellent sprouting tolerance and grain retention in the head at harvest. Moderately resistant to stripe, stem and leaf rust. Moderately susceptible–susceptible to crown rot. Resistant–moderately susceptible to common root rot, moderately resistant–moderately susceptible to yellow leaf spot and *Septoria tritici* blotch. Very susceptible to root-lesion nematode (*Pratylenchus thornei*). Some frost tolerance.	Wellings et al., (2005) and Amjad (2001) https://www.grainland.com.au/seed-sales-information
8	2001	Braewood	AH. Susceptible to yellow spot. Moderately resistant to rust. Medium height and suitable for production of high protein and high volume of breads and wanton dumpling skins.	Wellings et al. (2005), and Zaicou et al., (2010)
9	2007	Merinda	AH. Rust resistant. Most suitable to grow in NSW. Released as a Carinya and Janz replacement.	Wellings et al., (2005)
10	2012	Suntop	APH (Australian Prime Hard). Origin in northern NSW. Suitable to all environments across NSW and Queensland. Highest yields in independent trials. Best disease resistance variety. AGT Seeds.	Zeeshan et al., (2020) https://www.grainland.com.au/seed-sales-information

[1]References

Amjad, M. (2001). Yield and falling numbers of new wheat cultivars on the south coast of Western Australia.

Mares, D. J. (1983). Preservation of dormancy in freshly harvested wheat grain. *Australian Journal of Agricultural Research*, *34*(1), 33-38.

Parish, J. A., & Stone, R. F. (1965). Premium wheat in Western Australia. *Journal of the Department of Agriculture, Western Australia, Series 4*, *6*(9), 575-578.

Paull, J. G., Chalmers, K. J., Karakousis, A., Kretschmer, J. M., Manning, S., & Langridge, P. (1998). Genetic diversity in Australian wheat varieties and breeding material based on RFLP data. *Theoretical and Applied Genetics*, *96*(3), 435-446.

Reeves, J. T. (1963). Cereal variety trials, 1961-62. *Journal of the Department of Agriculture, Western Australia, Series 4*, *4*(1), 21-29.

Salman, H., Blazek, J., Lopez-Rubio, A., Gilbert, E. P., Hanley, T., & Copeland, L. (2009). Structure–function relationships in A and B granules from wheat starches of similar amylose content. *Carbohydrate Polymers*, *75*(3), 420-427.

Snape, J. W., Chapman, V., Moss, J., Blanchard, C. E., & Miller, T. E. (1979). The crossabilities of wheat varieties with Hordeum bulbosum. *Heredity*, *42*(3), 291-298. Wellings, C., Bariana, H., & Park, R. (2005). Cereal Rust Report.

Zaicou, C., Penny, S., Shackley, B., Ellis, S., Miyan, S., Dhammu, H., Shankar, M., & Sharma, D. (2010). Wheat variety guide 2010 Western Australia.

Zeeshan, M., Lu, M., Naz, S., Sehar, S., Cao, F., & Wu, F. (2020). Resemblance and difference of seedling metabolic and transporter gene expression in high tolerance wheat and barley cultivars in response to salinity stress. *Plants*, *9*(4), 519.

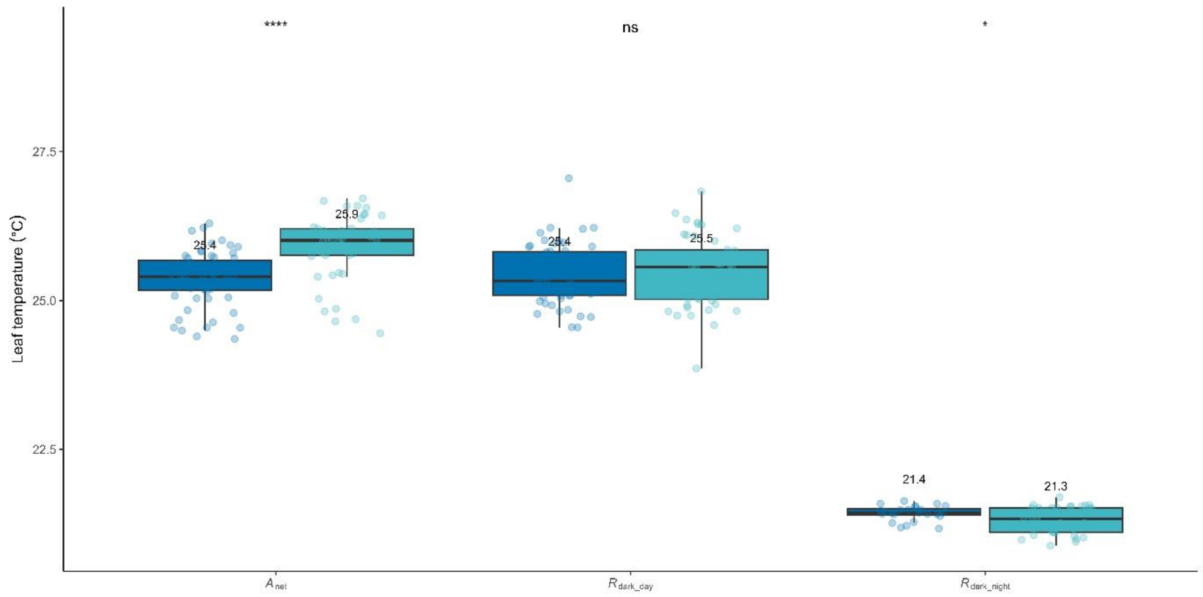

Supplementary Figure S1. Leaf temperatures during measurements of net CO_2 assimilation at 25 °C (A_{net}), daytime leaf dark respiration at 25 °C (R_{dark_day}) and night leaf dark respiration at 20 °C (R_{dark_night}) for control (blue boxplots) and high night temperature (green boxplots) plants. Central lines represent the medians; numbers indicate means across cultivars; points show cultivar-level observations. Boxes indicate interquartile ranges (IQR), whiskers extend to 1.5× IQR. Differences were assessed using Welch's two-sample t-tests. Significance annotations of paired temperature treatment comparisons based on Welch's t-test are indicated directly over boxplots as ***, * and ns for $P<0.001$, $P<0.05$ and not significant ($P>0.05$), respectively. n = 2-6.

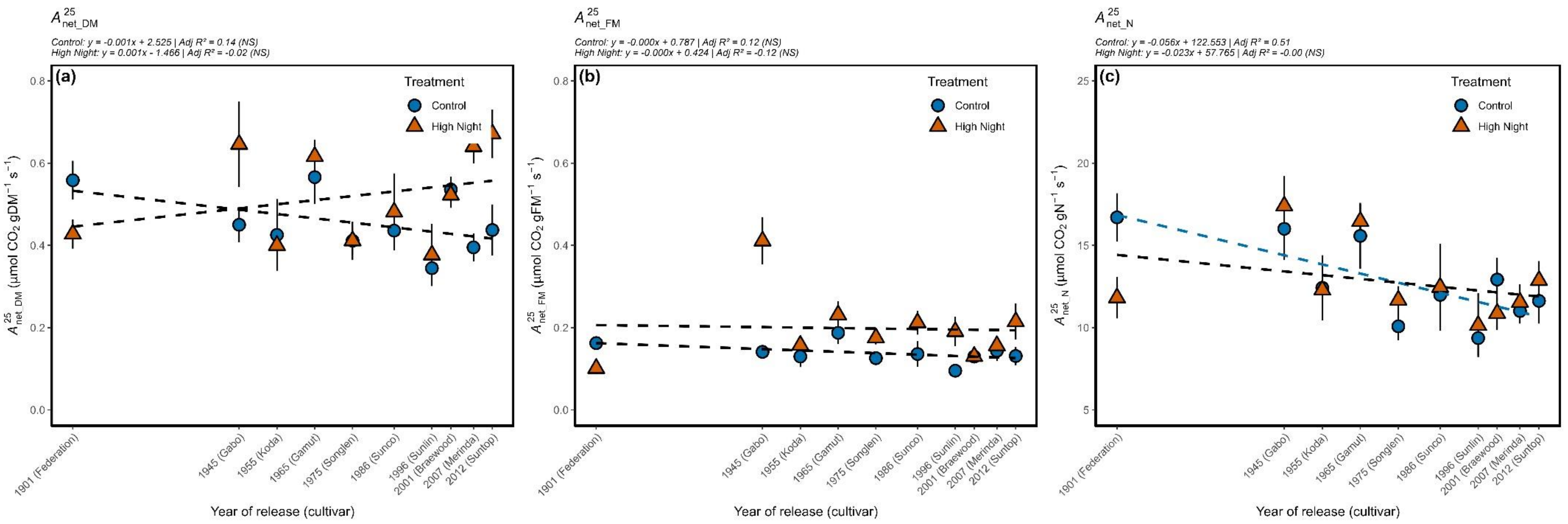

Supplementary Figure S2. Net rates of photosynthetic CO_2 assimilation per unit leaf (a) dry mass (A_{net_DM}); (b) fresh mass (A_{net_FM}); and (c) nitrogen content (A_{net_N}) measured at 25 °C of Australian wheat cultivars released between 1901 and 2012. Cultivars were treated to night temperatures of 12 °C (control, blue circles) or 22 °C (high night, red triangles). Adjusted R^2 are indicated at the top of each panel. Where regression slope is not significantly different from zero this is indicated by black dotted lines with NS next to the corresponding Adjusted R^2. Significant slopes are presented with treatment-specific colour. All measurements were done at a standard reference temperature of 25°C. Error bars are s.e.m. n = 2-6.

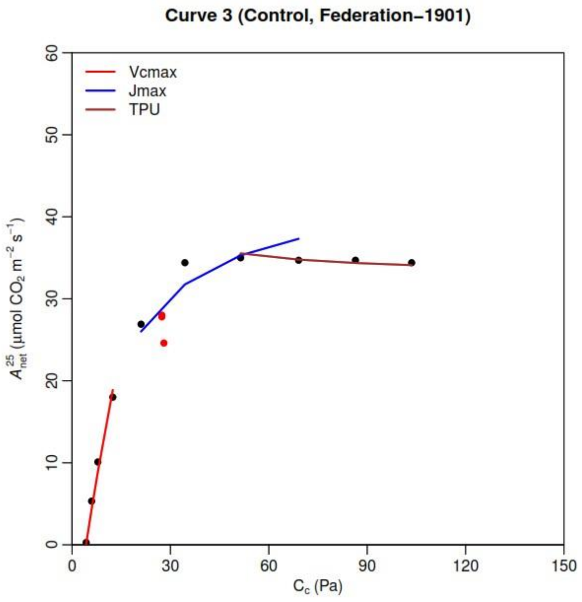

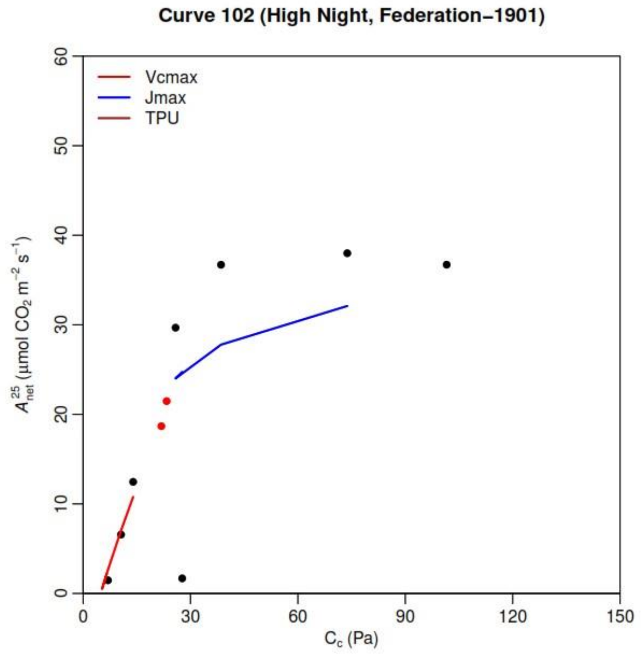

Supplementary Figure S3. Examples of good (top panel) and bad (bottom panel) plots of net rates of photosynthetic CO_2 assimilation measured at 25 °C ($A_{net,}$ µmol CO_2 m^{-2} s^{-1};) at different intercellular CO_2 concentration (C_i, Pa). C_i was converted to chloroplastic CO_2 concentration (C_c, Pa) based on von Caemmerer and Evans (2015). Black and red dots are actual observations. Data from good plots were used for determination of photosynthetic parameters (V_{cmax}, red lines; J_{1500}, blue lines; and TPU, brown lines) at 25 °C whereas those from bad plots were omitted from all subsequent analyses in this our study.

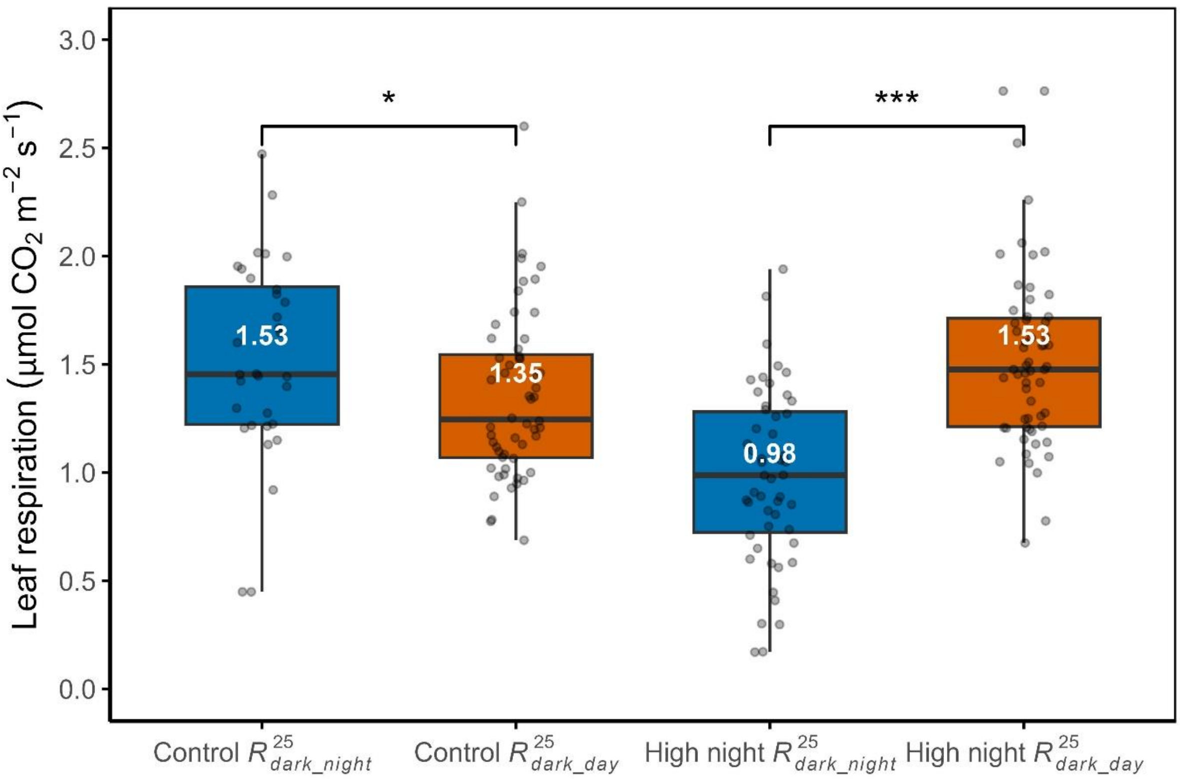

Supplementary Figure S4. Rates of leaf dark respiration at 25 °C either measured during the day ($R_{\mathrm{dark\_day}}$, red) at 25 °C or estimated from rates measured at 20 °C during the night ($R_{\mathrm{dark\_night}}$, blue) using the global polynomial respiration-temperature model of Heskel et al. (2016). Within each treatment, $R_{\mathrm{dark\_night}}$ was significantly higher than $R_{\mathrm{dark\_day}}$ under control conditions ($p < 0.05$), whereas the reverse pattern was observed under high night conditions ($p < 0.001$). Central lines represent the medians; bold white numbers indicate treatment means; points show cultivar-level observations. Boxes indicate interquartile ranges (IQR), whiskers extend to 1.5× IQR. Differences were assessed using Welch's two-sample t-tests. n = 2-6.
